# Optical coherence tomography features of neovascularization in proliferative diabetic retinopathy: a systematic review

**DOI:** 10.1186/s40942-020-00230-3

**Published:** 2020-06-29

**Authors:** Sara Vaz-Pereira, Tiago Morais-Sarmento, Raquel Esteves Marques

**Affiliations:** 1 Department of Ophthalmology, Centro Hospitalar Universitário de Lisboa Norte, EPE-Hospital de Santa Maria, Avenida Professor Egas Moniz, 1649-035 Lisbon, Portugal; 2 Department of Ophthalmology, Faculdade de Medicina, Universidade de Lisboa, Lisbon, Portugal; 3 Department of Ophthalmology, Hospital do Espírito Santo de Évora EPE, Évora, Portugal

**Keywords:** Diabetes mellitus, Diabetic retinopathy, Diagnostic imaging, Proliferative diabetic retinopathy, Retinal neovascularization, Tomography, optical coherence, Optical coherence tomography angiography

## Abstract

**Background:**

Diabetic retinopathy (DR) is a leading cause of blindness due to diabetic macular edema (DME) or complications of proliferative diabetic retinopathy (PDR). Optical coherence tomography (OCT) is a noninvasive imaging technique well established for DME but less used to assess neovascularization in PDR. Developments in OCT imaging and the introduction of OCT angiography (OCTA) have shown significant potential in PDR.

**Objectives:**

To describe the tomographic features of PDR, namely of neovascularization, both of the optic disc (NVD) and elsewhere (NVE), intraretinal microvascular abnormalities (IRMA), retinal nonperfusion areas (NPA), status of the posterior vitreous, vitreoschisis and vitreous and subhyaloid/sub-ILM hemorrhages.

**Data sources:**

Electronic database search on PubMed and EMBASE, last run on December 19th 2019.

**Study eligibility criteria, participants and interventions:**

Publications assessing OCT and/or OCTA findings in PDR patients. All study designs were allowed except for case-reports, conference proceedings and letters.

**Study appraisal:**

Newcastle–Ottawa Scale for observational studies was used for purposes of risk of bias assessment.

**Results:**

From the 1300 studies identified, 283 proceeded to full-text assessment and 60 were included in this comprehensive review. OCT was useful in detecting NVD and NVE, such as in characterizing disease activity and response to laser and/or anti-VEGF therapies. The absence of posterior vitreous detachment seemed determinant for neovascular growth, with the posterior hyaloid acting as a scaffold. OCTA allowed a more detailed characterization of the neovascular complexes, associated NPA and disease activity, allowing the quantification of neovessel area and flow index. However, changes in OCTA blood flow signal following local therapies did not necessarily correlate with structural regression. Widefield and ultra-widefield OCTA were highly sensitive in the detection of PDR, adding value to disease staging and monitoring. Compared to fluorescein angiography, OCTA was more sensitive in detecting microvascular changes indicating disease progression.

**Limitations:**

Publication languages were restricted. Most included studies were observational and non-comparative. Risk of bias regarding case representativeness.

**Conclusions:**

OCT-based retinal imaging technologies are advancing rapidly and the trend is to be noninvasive and wide-field. OCT has proven invaluable in diagnosing, staging and management of proliferative diabetic disease with daily application in clinical and surgical practices.

## Background

Diabetes mellitus affected an estimate of 463 million people worldwide in 2019 and this number is projected to rise to 578 million by 2030 and 700 million by 2045 [1]. Diabetic retinopathy (DR) is its leading microvascular complication and a major cause of blindness [2, 3]. Vision-threatening DR is due to diabetic macular edema (DME) or proliferative DR (PDR) [2, 3]. A pooled meta-analysis estimated a global prevalence of 7.5% for PDR [2] meaning approximately 35 million diabetic people had PDR in 2019 and by 2030 and 2045 around 43 and 53 million will be affected, respectively. The high impact and global burden of PDR urge the need to continue researching on diagnostic and treatment modalities.

The hallmark of PDR is the presence of retinal (NVE) or disc (NVD) neovascularization [4, 5] and color fundus photography (CFP) and fluorescein angiography (FA) have been the most relevant imaging techniques used in the last decades [6–9]. In recent years, there have been significant developments in noninvasive imaging technologies [10–12]. Optical coherence tomography (OCT) was first introduced in ophthalmology in 1996, becoming standard of care for macular disease in 2005 [13]. In DR, it has been primarily used in DME assessment [9] and in 2013 its usefulness in PDR was first reported [14]. OCT allows the evaluation of diabetic neovascularization in the earliest stages and to recognize associated vitreoretinal interface changes [14–18], but information about vessel structure and blood flow cannot not be obtained [19, 20], limiting its application in evaluating disease progression and treatment response [18, 21]. In 2014, commercial OCT angiography (OCTA) was introduced [13] and in the last years there has been a significant number of publications demonstrating proliferative diabetic changes [22–28], some even using widefield (WF) imaging [29–32]. Combining OCT with the surgical microscope also aided in the immediate evaluation of vitreoretinal structures and in identifying surgical planes, helping in membrane delamination/segmentation, a crucial step in diabetic tractional retinal detachment (TRD) surgery [12, 33–35].

In this review, we analyzed all studies using OCT and/or OCTA so as to perform a comprehensive description of the tomographic features of PDR, namely of (1) NVD and (2) NVE. As secondary outcomes we documented information regarding (3) intraretinal microvascular abnormalities (IRMAs); (4) retinal nonperfusion areas (NPAs); (5) status of the posterior vitreous; (6) vitreoschisis; and (7) vitreous and subhyaloid/sub-internal limiting membrane (ILM) hemorrhages. OCT-based retinal imaging technologies are advancing rapidly and the trend is to be noninvasive and widefield. Therefore, it is important to revise noninvasive PDR imaging for future application in daily clinical and surgical practices.

## Methods

We performed an electronic database search on PubMed and EMBASE, last run on December 19th, 2019 and adopted PRISMA (Preferred Reporting Items for Systematic Reviews and Meta-Analyses) guidelines using a PRISMA checklist (Additional file 1). Search design meant to reach all studies assessing OCT and/or OCTA findings in PDR patients—features of neovascular complexes (NVCs), IRMAs, NPAs, status of the posterior vitreous and subhyaloid/sub-ILM hemorrhages—published in English, French, Spanish or Portuguese. All designs and publication types were accepted, except for case-reports, conference proceedings and letters. Studies based on time-domain OCT were also excluded, considering its lower resolution and higher artifact profile. No restrictions existed on age, diabetes type, metabolic status or follow-up. A detailed search strategy is provided in Additional file 2.

All papers were screened through title and abstract by two independent reviewers, proceeding to full-text assessment when eligible. Article selection was based on three criteria: theme within scope of review; assessment of outcomes of interest; and appropriate methodological quality. The latter was assessed through the Newcastle–Ottawa Scale for observational studies [36]; this tool was modified to assess non-comparative studies as well, removing all topics on comparability. The articles’ reference list was hand-searched for additional studies.

Data extraction was performed in duplicate by study authors, guaranteeing double verification as a way to minimize reporting errors. The Newcastle–Ottawa Scale for observational studies was used for purposes of risk of bias assessment, performed by 2 authors on the individual study level. This tool was modified to assess non-comparative studies as well, removing all items on group comparability. Reviews were assessed considering the quality of their included studies. Disagreements concerning inclusion or methodological appropriateness were solved by consensus or a third author.

## Results

**Fig. 1:**
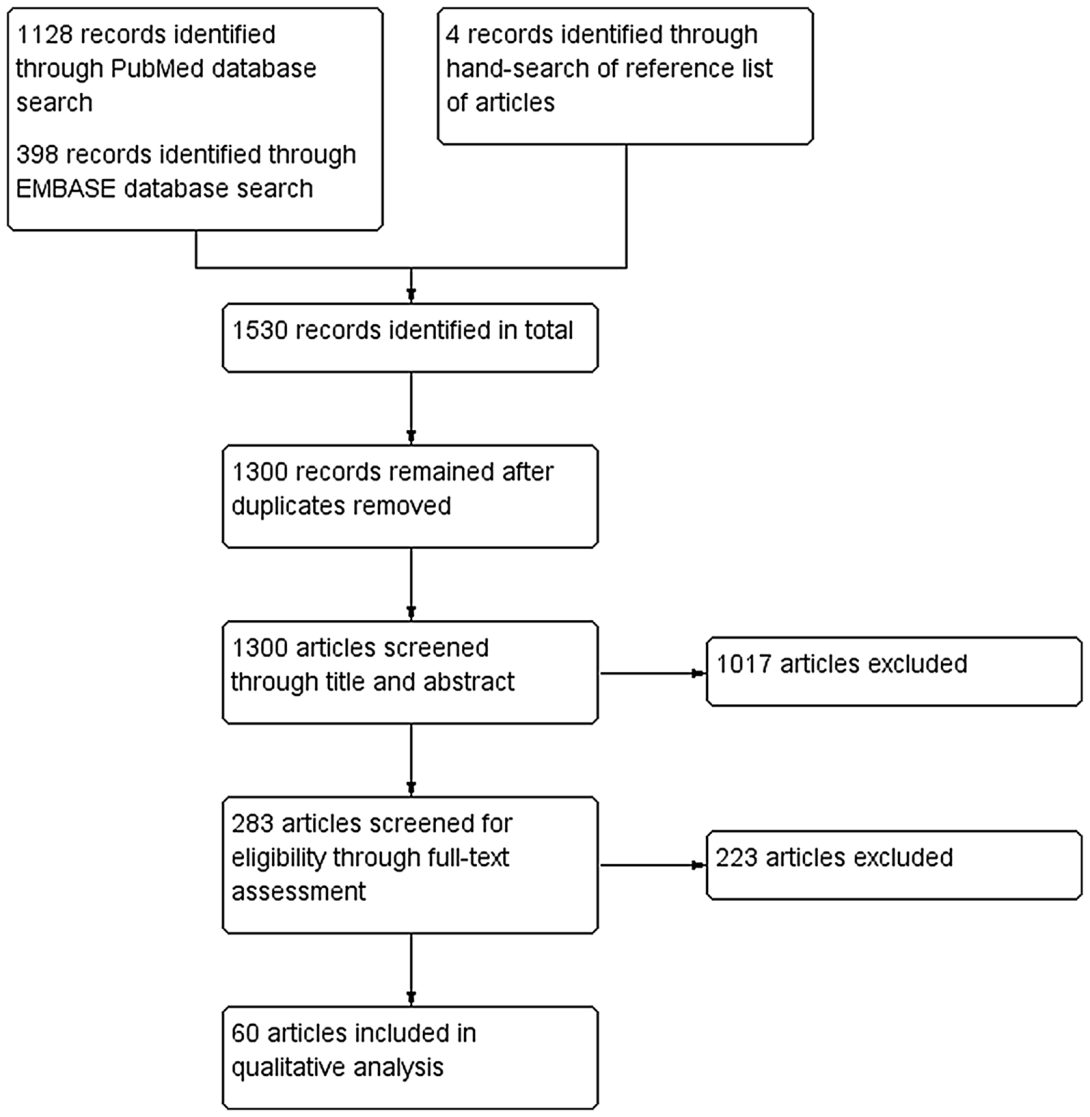
PRISMA study flow diagram

Our search identified 1530 studies; after duplicates removed, 1300 studies were screened for inclusion through title and abstract, with 283 proceeding to full text-assessment, and 60 included in this qualitative systematic review (Fig. 1). A summary of included studies is provided in Additional file 3.

The studies included were mostly observational and non-comparative, with the following designs presented in decreasing frequency: observational non-comparative series/cohort—26 papers; review—17; non-comparative studies assessing retinal changes following local treatment (either observational or interventional)—11; comparative studies (vs. healthy controls)—5; secondary analysis of previously published prospective trials—1.

Overall, included studies were classified as having a moderate-to-good methodological quality. Risk of bias was low regarding both case selection/definition and ascertainment of outcomes, as these were depicted in imaging exams, providing solid documentation of disease and outcome status. On the other side, a more significant risk of bias was assessed regarding the representativeness of cases, as only a minority of studies reported a systematic or consecutive inclusion of cases.

### Tomographic features of NVCs

#### NVD

**Fig. 2:**
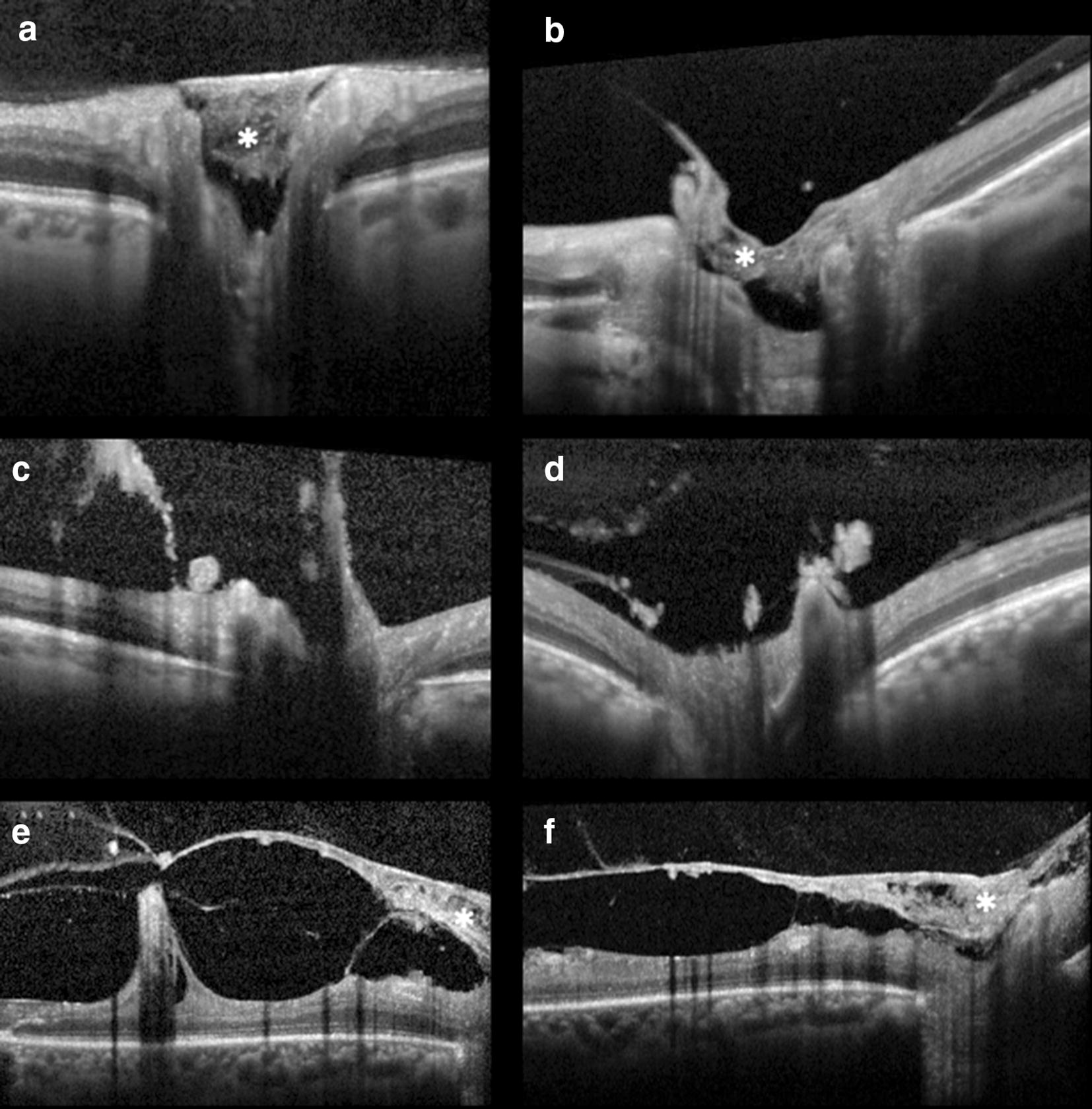
Examples of NVD on structural SD-OCT. **a**, **b** NVDs (asterisk) as hyperreflective tissue sitting on the disc with an attached posterior hyaloid. **c**, **d** NVD protruding from the disc into the vitreous with posterior hyaloid breaching, in **c** note the detached hyaloid. **e**, **f** Depict advanced NVDs with thick fibrovascular tissue (asterisk) protruding from the disc and growth along the posterior hyaloid, which serves as scaffold, into the peri-papillary area and macular traction

Thirty-seven studies described NVD characteristics [9, 12, 14, 15, 18–23, 25–32, 37–55]. Tomographic evaluation of NVDs using structural spectral-domain OCT (SD-OCT) was first reported in 2013 by Cho et al. [14], who identified NVDs as hyperreflective tissue sitting or protruding from the optic disc into the vitreous, whether the posterior hyaloid (PH) was attached or detached, respectively (Fig. 2). Muqit et al. [15] further described subclinical NVDs as sitting over the disc and attached to the outer hyaloid and early NVDs as looped structures containing small hyporeflective spaces. More advanced NVDs consisted on thick tissue protruding from the disc that grew axially along the PH and extended into the peri-papillary ILM surface (Fig. 2) [15, 37]. Absence of posterior vitreous detachment (PVD) on the disc was more frequent among active NVDs. These originated outside the physiological cupping and grew along the PH which served as scaffold. Occasionally, there was breaching of the PH and growth into the vitreous cavity [15, 17, 18, 48]. After laser treatment, NVDs were seen to regress with involution of the vitreous projections, and larger NVDs were described as free-floating in the vitreous due to tractional avulsion [15].

**Fig. 3:**
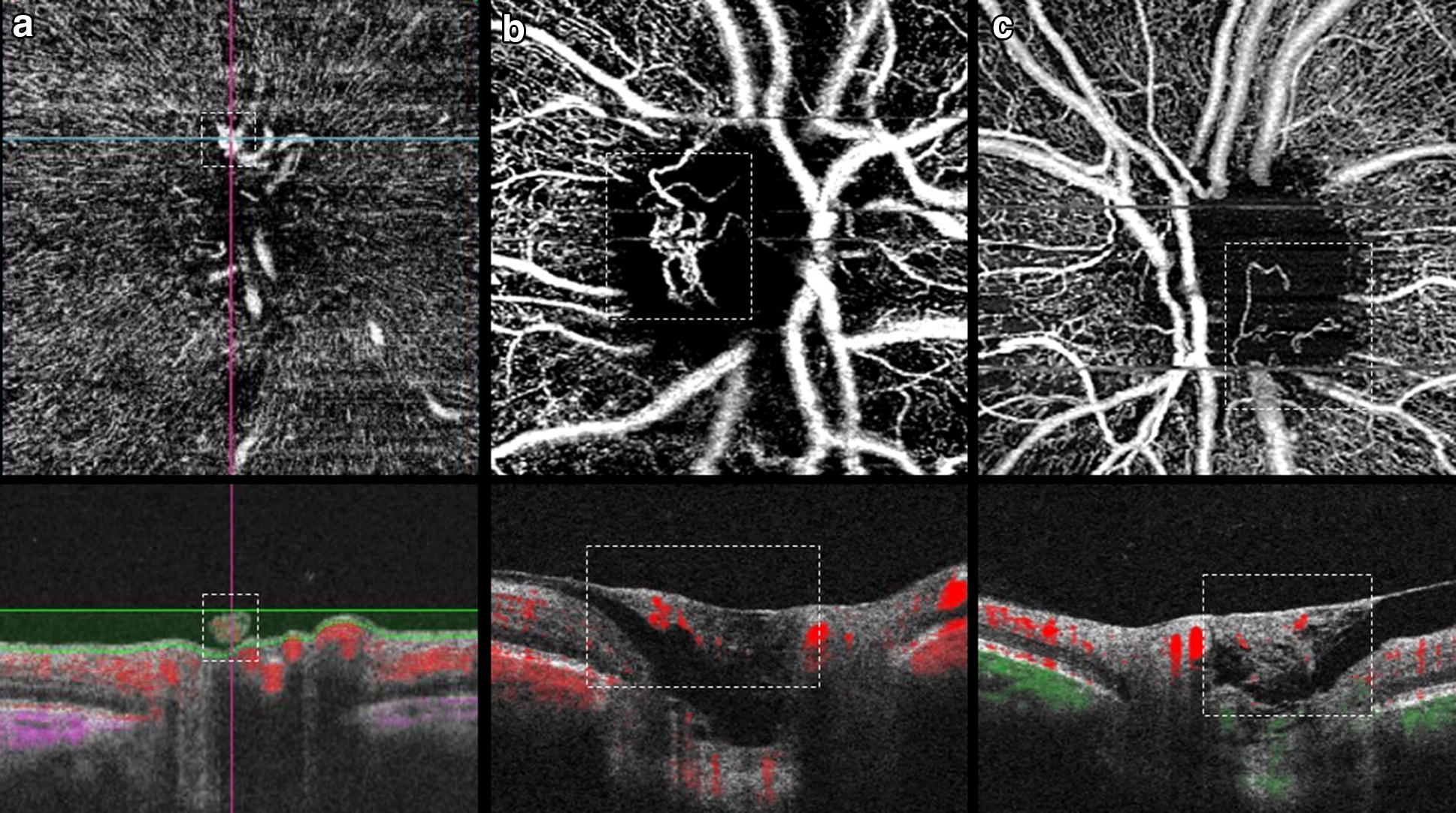
Examples of NVD using OCTA. **a** Swept-Source OCTA showing a small early NVD in the en-face angiogram (dashed line, top), which corresponded in the OCT B-scan to a structure above the disc with positive flow signal (dashed line, bottom), indicating active disease. **b** SD-OCTA demonstrating an active NVD with irregular new vessels on the en-face image (dashed line, top) and flow signal in red on the OCT B-scan (dashed line, bottom). **c** Example of an NVD with pruning and little branching on the en-face image (dashed line, top), with fibrotic tissue and minimum flow signal on the co-registered B-scan (dashed line, bottom)

More recently, OCTA identified the vascular structure of NVDs by the presence of blood flow signal in the en-face OCT angiogram, which corresponded in the OCT B-scan to structures with positive flow signal above the optic disc or peripapillary retinal surface (Fig. 3) [19, 22, 23, 26–28, 38–45, 52]. They originated from the retinal vein, artery or choroid and arose from bending vessels inside or near the optic disc rim, mostly in the upper temporal sector [21, 25]. Quantification of vessel area/flow index was useful to determine the extent and activity of NVDs [23, 41–43, 46] and their changes after anti-VEGF/laser treatment [23, 26, 27, 29, 31, 39, 43, 44, 46, 51]. Alteration in NVD blood flow in OCTA did not necessarily translate into structural regression [23].

Ishibazawa et al. [28] introduced the concept of exuberant vascular proliferation (EVP), as the proliferation of irregular smaller-caliber vessels in more active disease, correlating with more active leakage in FA than NVCs without EVP. Pruning and regression in EVP with reduction in NVD flow area was documented after panretinal photocoagulation (PRP) [28]. Various studies found that OCTA and WF-OCT were able to define all NVDs [20, 39, 46, 47], with a superior visualization when compared to FA [20]. B-scan OCT had an 82.9% NVD detection rate [27].

Elbendary et al. [39] using swept-source OCT (SS-OCT) documented 3 NVD patterns: (1) active NVDs, with a medium reflectivity vascular component originating from the disc, sometimes showing an anastomosing branching network with EVP in the en-face OCTA, and with blood flow signal in the co-registered B-scans; (2) active NVDs, with medium–high reflectivity fibrovascular elements related to an incompletely detached PH, with little branching in the OCTA but with some flow signal; and (3) NVDs with a hyperreflective fibrotic component growing along a thicked and incompletely detached PH, with no flow signal in the en-face OCTA and minimum flow signal on the B-scan.

#### NVE

**Fig. 4:**
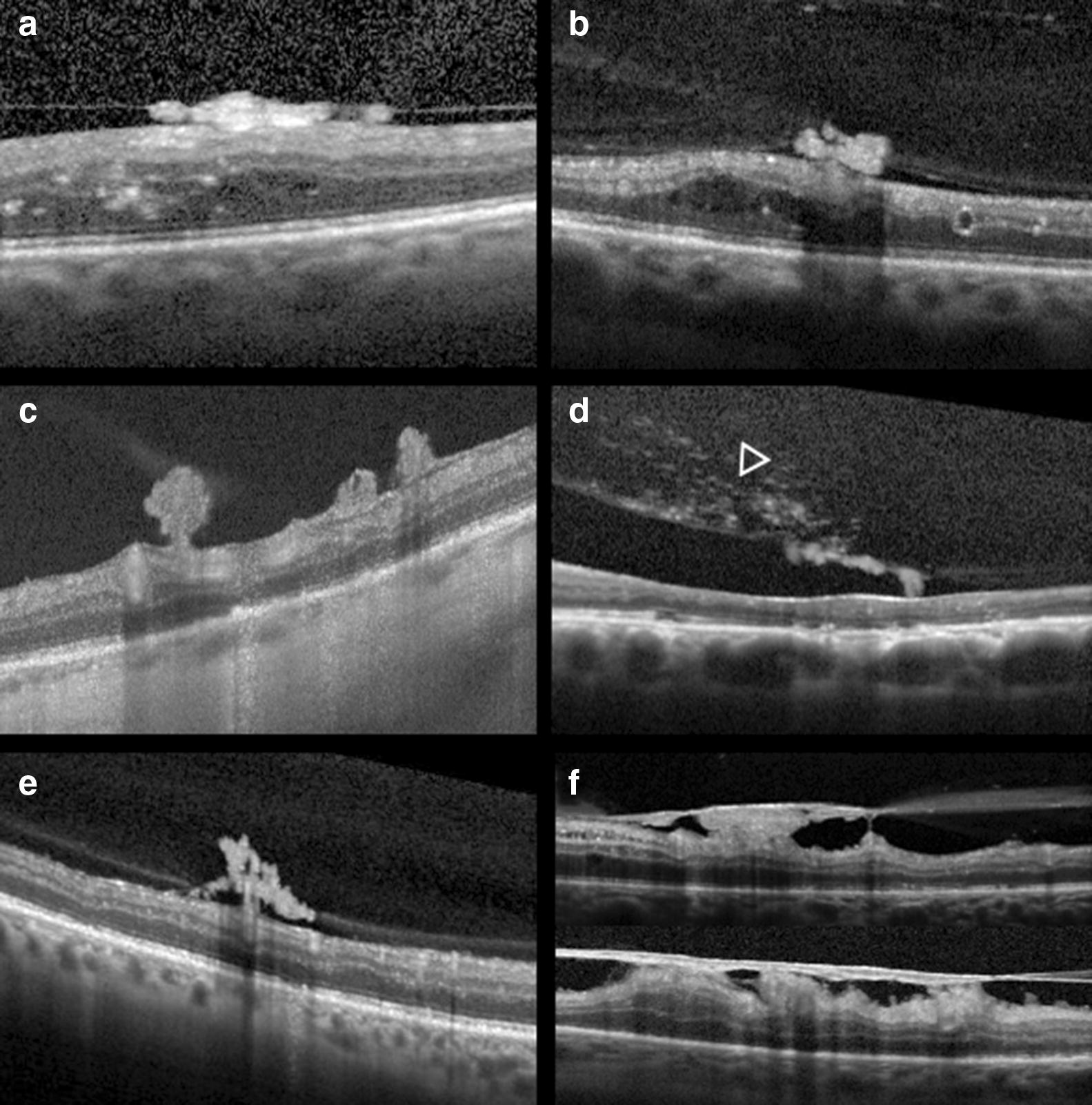
Examples of the morphology of NVE on structural SD-OCT. **a**, **b** Flat NVE confined to the posterior hyaloid. **c**, **d** Forward configuration into the vitreous, in **d** note the hyperreflective dots in the vitreous (arrowhead) corresponding to localized vitreous hemorrhage. **e** Flat lesion growing along the posterior hyaloid with forward extensions and vitreous invasion. **f** Tabletop NVEs tethered to the retina by multiple retinal adhesions/pegs

Forty-two studies reported NVE features [14–19, 21, 22, 24–31, 35, 37–39, 41, 42, 44, 45, 47, 49–54, 56–66]. In structural OCT, NVEs presented as homogenous hyperreflective loops breaching the ILM and protruding into the vitreous with posterior retinal shadowing (Fig. 4) [14–16, 18, 19, 37, 50]. These complexes arose from the outer plexiform layer and extended through the inner retinal layers, penetrated the ILM and attached to the PH [15]. As seen in NVDs, PH served as scaffold for NVE development and in most cases is attached or partially detached and tethered to neovascular tissue [16–18]. Using SD-OCT, NVEs have been proposed to develop in 3 stages: I—disruption of ILM; II—horizontal growth along ILM and III—multiple breach of PH and linear growth [16]. According to their morphology they have been classified by Vaz-Pereira et al. [17] as (1) flat, when confined to the PH face; (2) forward when lesions showed PH traversal and (3) tabletop when NVCs where displaced anteriorly by vitreous traction but tethered to the retina (Fig. 4). NVEs were also classified according to location in (1) above the ILM and (2) below the ILM types [56] based on their intraretinal component [15, 67, 68]; nonetheless most use the histopathology definition of NVE, where a breach of the ILM is a requisite [4, 16, 69, 70].

**Fig. 5:**
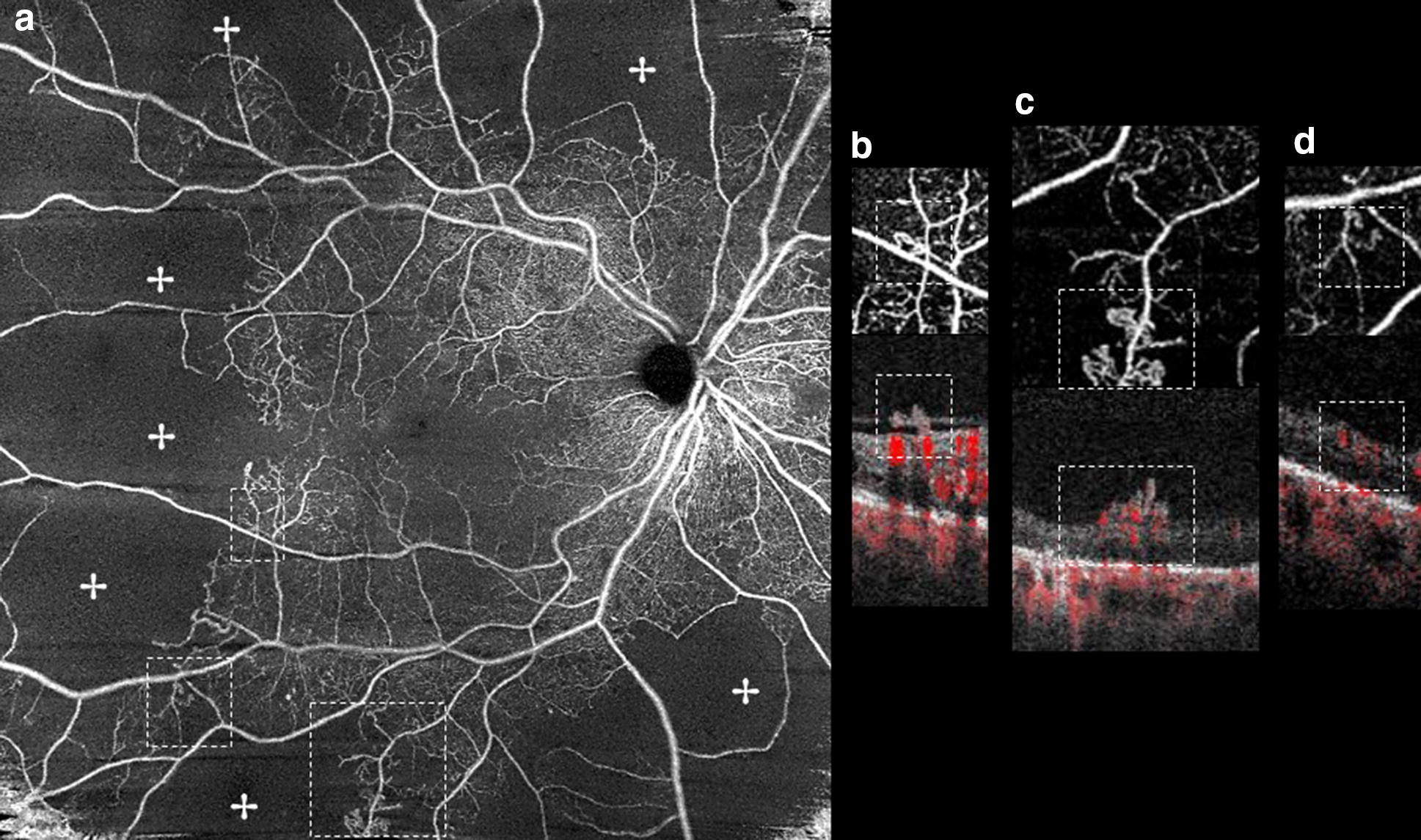
**a** En-face SD-OCTA 8 × 8 mm montage showing significant areas of retinal nonperfusion (✢). Magnification of top (**b**) and bottom (**c**) annotated area in **a** shows small neovascular complexes on the en-face angiogram breaching the ILM and with positive flow signal in the structural B-scan (within dashed lines), in accordance with active NVEs. Note than in (**d**), left annotated area, the microvascular abnormality depicted in the en-face angiogram does not breach the ILM in the co-registered B-scan and the flow signal is only intraretinal, in accordance with IRMA

With OCTA, by combining the en-face angiograms with B-scans, NVEs appeared as irregular masses of vessels with positive flow signal above the ILM, distinguishing them from microaneurysms and IRMA (Fig. 5) [9, 21, 22, 24–31, 39, 41, 42, 44, 51–53, 59–65]. Structural OCT and OCTA B-scans were found to detect 100% of NVEs while the en-face OCTA, CFP and biomicroscopy detected 72.7% of cases [27]. A study using WF-OCTA evaluated the distribution of NVCs and found NVEs more prevalent superotemporally [32].

Pan et al. [25] classified NVE based on origin and branching pattern on OCTA: type 1 (most common) originated from a major retinal vein adjacent to NPA, breaching the ILM at a single location and forming many branches after attaching to the PH; type 2 originated from the capillary within the NPA and breached the ILM at multiple locations; and type 3 developed from pre-existing IRMAs within NPA and most of the structure was intraretinal, except for some tips that breached the ILM to form neovascularization. The EVP concept introduced by Ishibazawa [28] was also applied to NVEs and changes in NVE area/flow signal were also observed after PRP or anti-VEGF treatment, establishing OCTA as a valuable tool to evaluate and monitor PDR [28, 31, 41, 42, 44, 51, 56, 63, 65].

### Shared features of NVCs

The presence of vitreous hyperreflective dots in SD-OCT was more significant in active PDR, while the presence of epiretinal membrane, inner retinal tissue contracture, vitreous invasion and vitreous protrusion was more associated with quiescent disease [18]. Using OCTA, EVP was more frequent in treatment-naïve NVCs—thus EVP should be considered an active sign of neovascularization [28].

When comparing imaging modalities, structural OCT and B-scan OCTA had the best detection rate for new-onset NVCs, but B-scan OCTA was superior for follow-up, due to the persistence of the NVC tissue on OCT [27]. Also, WF and ultra-WF (UWF) OCTA demonstrated increased value in NVC imaging. When screening patients previously classified as non-proliferative DR, WF-OCTA identified NVCs in 15% of patients, while conventional OCTA scans could only detect 7 and 0% NVCs [71]. WF-SS-OCTA had sensitivity and specificity values of 79% and 96% in the detection of NVCs—similar to FA [58] and wide-angle OCTA sensitivity of 100% and specificity of 97% [61]. Compared to UWF FA, SS-OCTA was more sensitive in detecting microvascular changes indicating possible disease progression [27, 31] with some authors suggesting WF-OCTA as the single preferred imaging strategy for PDR detection and monitoring [29, 31, 44, 52].

### Tomographic evaluation of IRMA

Nineteen studies mentioned IRMA characteristics [14, 16, 19, 21, 22, 24–26, 30, 31, 38–41, 54, 56, 59, 65, 72]. These were present in 25–85% of examined diabetic eyes [14, 30] and were described using OCT as areas of focal/multifocal, tortuous, dilated looped vascular segments [21, 22, 25, 26, 30, 38, 40, 59, 65]. IRMA originated from and drained into a retinal venule, arising from the inner plexiform (67%), ganglion cell (17%) or nerve fiber layers (17%) [19, 25]. These hyperreflective structures were intraretinal, frequently extending across more than one layer [40] and without ILM breach [14, 16, 21, 24–26, 30, 31, 38–41, 59, 65]. However, focal areas of outpouching of the ILM [9, 14, 16, 40] were sometimes observed—even in these cases, the ILM and the PH were intact. Lee et al. [16] defined IRMA with outpouching of ILM as stage 2 IRMA, while the remaining were stage 1. Similarly, the OCTA flow overlay depicted mild-moderate intraretinal flow possibly outpouching and distorting ILM contour, without breaching through the ILM or PH (Fig. 5) [59]. These outpouching IRMA seemed to have relative increased vascularity on flow overlay, when compared to nonoutpouching IRMA and surrounding normal vasculature [59]. The ability to breach through the ILM to differentiate into NVE seemed to be exclusive to sea-fan-like IRMA [25].

IRMA were consistently identified adjacent to NPAs [21, 22, 25, 26, 30, 38–40, 59]. Pan et al. [25] measured the associated NPA, averaging 11.48 ± 4.92 mm^2^, while Schaal and colleagues [30] linked IRMA to NPA occupying ≤ 1/4 disc area—in both cases, these areas were smaller than the associated with NVE. One series described NVCs directly adjacent to IRMA in 50% of the studied eyes, reinforcing the concept of nonperfused retinal environment [24].

SD-OCT revealed that 20% of clinically diagnosed IRMA were actually misclassified NVE, with ILM and PH breaching; one of these complexes did not leak on FA, precluding its correct classification [16]. As for SS-OCTA, the interrater agreement for the detection of IRMA was good (k = 0.7) [30]. Of the definite IRMA detected with this technology, only half were imaged in CFP (ETDRS protocol); conversely, all IRMA detected on CFP were imaged with SS-OCTA, portraying a higher sensitivity for IRMA detection with OCT [30].

In angiographic studies, vessel wall staining was noted [22], but prominent fluorescein leakage was mostly excluded—distinguishing IRMA from NVE [14, 19, 40, 56]. However, in one series, minimal late fluorescein leakage was observed in ≈ 7% of IRMA—but the amount of leakage could not be related to any of the OCTA imaging characteristics [59]. A low agreement was reported between fluorescein leakage on FA and OCT discrimination of NVE vs. IRMA (k = 0.25); in this paper, the authors depicted a case-study of a patient with 4 IRMA severely leaking on FA, of which most breached the ILM to become NVE during follow-up [16].

When compared to FA, WF-OCTA demonstrated a sensibility of 92% and a specificity of 99% to differentiate between IRMA and NVE, with a positive and negative predictive value of 96% and 97%, respectively [59]. Compared to adaptive optics scanning laser ophthalmoscopy, SD-OCT showed a decreased sensitivity detecting ≈ 90% of total IRMA [72].

### Additional findings

#### Retinal nonperfusion areas

NPAs represent capillary occlusion and are regarded as ischemic areas [11, 23, 38, 40]. Using OCTA, NPAs were identified as areas of absent signal because of low capillary perfusion or dropout (Fig. 5) [22, 23, 38, 40, 42]. Frequently, delineation of these areas was superior to FA [22, 23, 40], with a sensitivity of 98% and specificity of 82% for wide-angle OCTA [61]. NPAs could be quantified [29, 30, 60] and PDR patients were found to have a significant lower capillary density compared to non-PDR patients and increased and irregular FAZ [45, 60]. Also, IRMAs and NVCs were more frequently associated with NPAs [23–25, 30, 38–42, 58, 65, 66, 73].

#### Hemorrhage

Bleeding from NVCs may cause vitreous or subhyaloid/sub-ILM hemorrhages. These result from increased permeability of active NVCs leading to extravasation of sanguineous components or from tractional forces exerted in NVCs which are adherent/tethered to the PH [15–18, 25, 50, 74]. Vitreous hemorrhage was documented on OCT as vitreous hyperreflective opacities/dots, which were positively associated with active NVCs (Fig. 4) [15, 16, 18, 50, 74]. Subhyaloid/sub-ILM hemorrhages consist of hyperreflective material delineated by a thin reflective band anterior to the retina and posterior shadowing from masking of the OCT signal by the hemorrhage [15, 37, 50, 75].

#### Vitreoschisis

Vitreoschisis corresponds to splitting of the posterior vitreous cortex and is a consequence of anomalous PVD, where a strong vitreomacular adhesion splits the posterior vitreous, leaving an outer layer attached to the retina while the remaining vitreous collapses anteriorly [17, 37, 50].

It is represented in OCT as multilayered hyperreflective bands of the posterior vitreous cortex, separated by an optically clear hyporeflective space [17, 37, 50, 74]. In one study, in 62% vitreoschisis cases, NVCs traversed the PH face to invade the schisis cavity, which may explain patterns of neovascular growth [17]. Vitreoschisis can be identified between NVCs anchorage points and may be associated with tractional retinoschisis, vitreomacular traction and/or TRD [17, 37, 74]. Vitreoschisis can complicate PDR by causing additional traction on NVCs, which may contribute to bleeding and complicate vitreoretinal surgery [37, 74].

#### Traction

NVCs-induced traction might lead to hemorrhaging, avulsed retinal tissue, TRD, cystoid edema and/or retinoschisis. These findings were documented tomographically as vitreous hyperreflective material, retinal disorganization with a concave configuration and adhesions/pegs, retinal hyporeflective cystic spaces and retinal layer splitting, respectively (Fig. 2) [14, 15, 18, 19, 37, 74, 76]. Considering its high-resolution and ability for microstructural delineation of tractional NVCs, OCT has proven invaluable in identifying the cleavage plan in retinal surgery when used preoperatively and intraoperatively, resulting in safer surgeries [12, 15, 17, 19, 27, 35, 37].

## Discussion

OCT is an established noninvasive technology mandatory in the management of DR. Although widely used to evaluate DME, recent studies have demonstrated its utility in assessing PDR. The first reports on diabetic NVCs were based on histopathology [4, 16, 68, 70], but OCT has shed significant light on NVC development, growth and response to treatment. OCT provides images in real time and in vivo—as an optical biopsy [13].

Structural OCT can identify NVCs, IRMAs and associated vitreoretinal changes [14–18, 37], but is limited by not recognizing blood flow. NVDs can be observed as tissue sitting or protruding from the disc. Regarding NVEs, it is important to distinguish them from IRMAs as the presence of neovascularization is the hallmark of PDR and indicates more severe disease, with implications in treatment and prognosis [4, 5]. IRMAs may be precursors of NVE, but are a definite risk factor for PDR [16, 24, 68].

The status of the posterior vitreous is also significant in PDR evaluation as the presence of PVD is believed to be protective for the development of PDR [16–18, 77]. Moreover, it is known that diabetic vitreopathy is responsible for a strong adhesion between the vitreous and retinal vessels, resulting in an incomplete anomalous PVD and vitreoschisis, with the PH acting as scaffold for newly growing NVCs [17, 37, 78]. Vitreoschisis can mimic complete PVD, but is important to distinguish them since prognosis is worse in vitreoschisis, possibly leading to bleeding and/or TRD [17, 78]. Tractional forces exerted on NVCs attached/tethered to the PH result in hemorrhaging, which is observed as vitreous hyperreflective dots or as hyperreflective material between the PH/ILM and the retina [15–18, 25, 50, 74]. Indeed, vitreous hyperreflective dots are associated with NVC activity either by representing bleeding and/or inflammatory cells [15, 16, 18, 50, 74, 79]. The study of the vitreoretinal interface is also valuable when preparing for surgical management. Incomplete PVD with or without retinal traction or vitreoschisis may increase the risk of intraoperative bleeding, for which the retinal surgeon must plan in advance [14, 15, 17, 35, 37, 78]. To date, few studies have addressed the use of intra-operative OCT and it will be interesting to see in the next few years if tomography will claim its place in the operating room [33–35].

OCTA brought a major advance as it clearly demonstrates microvascular changes by showing the vascular structure in the en-face image and the flow signal in the co-registered B-scans. These features are important to differentiate NVEs from IRMAs, to establish and quantify the presence of both NVD and NVEs, monitor treatment response and noninvasively evaluate other findings such as NPAs [19–32, 38–45, 47–49, 51, 57–63, 71]. When compared to biomicroscopy and CFP, OCT and OCTA were more reliable in identifying and distinguishing IRMA and NVCs [27]. OCTA compared to FA can perform better in the study of NPAs [22, 23, 40] and microvascular abnormalities as they may not leak and/or be obscured by fluorescein leakage [22, 23, 40]. In this context, UWF and WF-OCTA may be of particular interest, as a structural and functional wide view of the retina is provided non-invasively [29–32, 44, 52, 61, 71]. The safety of these complementary tests allows a more frequent imaging follow-up, probably achieving a tighter control of the retinal vascular status.

Having considered the advantages of tomography studies and despite its recognizable worth, we should keep in mind OCTA is an expensive technology, still unavailable in many ophthalmology practices, especially in its widefield and ultra-widefield variants, and the en-face image must be evaluated with the corresponding structural B-scan. As so, making the most out of available standard OCT techniques is still necessary.

The main strengths of our study are its rigorous methodology as we performed a systematic comprehensive search of articles using OCT and/or OCTA to document features of PDR and the features observed were similar and reproducible even when different OCT and/or OCTA machines were used. Limitations include (1) language restriction as we have only evaluated papers in English, French, Spanish or Portuguese; (2) study design, as most studies were observational case-series or non-comparative and cross-sectional, (3) risk of bias appraised as meaningful regarding case representativeness, as most studies did not describe a systematic/consecutive case selection. This last limitation could set barriers to the widespread application of our findings; however, considering the inclusion of a vast array of papers published on the topic, we believe we covered all existing literature on the tomographic findings of PDR.

## Conclusions

Modern OCTs enhanced the ability to detect and monitor PDR in a safe and non-invasive way.

In this review, we analyzed studies using OCT and/or OCTA to evaluate neovascularization in PDR, anticipating our comprehensive summary of results will help in the current and future real-world assessment of these patients. As knowledge and experience increase, OCTA and WF-OCTA have been proving their added benefit not only in NVC detection, but also in further characterizing NPAs and microvascular abnormalities—stratifying the odds for DR progression towards high-risk stages. As so, these imaging modalities will definitely establish their value in the clinical setting. This review intended to be one of the steps in this process by sharing and summarizing information for tomography users.

## Supplementary information

**Additional file 1: Table S1.** PRISMA checklist.

**Additional file 2: Figure S1.** Detailed search strategy.

**Additional file 3: Table S2.** Summary of included studies.

## Data Availability

The data is available from the corresponding author upon reasonable request.

## Abbreviations

CFP: Color fundus photography
DME: Diabetic macular edema
DR: Diabetic retinopathy
EVP: Exuberant vascular proliferation
FA: Fluorescein angiography
FAZ: Foveal avascular zone
ILM: Internal limiting membrane
IRMA: Intraretinal microvascular abnormalities
NPA: Retinal nonperfusion areas
NVC: Neovascular complex
NVD: Neovascularization of the disc
NVE: Neovascularization elsewhere
OCT: Optical coherence tomography
OCTA: Optical coherence tomography angiography
PDR: Proliferative diabetic retinopathy
PH: Posterior hyaloid
PRP: Panretinal photocoagulation
PVD: Posterior vitreous detachment
SD-OCT: Spectral-domain optical coherence tomography
SS-OCT: Swept-source optical coherence tomography
SS-OCTA: Swept source optical coherence tomography angiography
TRD: Tractional retinal detachment
VEGF: Vascular endothelial growth factor
VH: Vitreous hemorrhage
UWF: Ultra-widefield
WF: Widefield
