# Supplementary material for: Optical coherence tomography features of neovascularization in proliferative diabetic retinopathy: a systematic review
Source: Int J Retina Vitreous. 2020 Jun 29;6:26. doi: 10.1186/s40942-020-00230-3 (PMC7322867; doi:10.1186/s40942-020-00230-3)
Supplement: Supplementary file 2 — Additional file 2: Figure S1. Detailed search strategy. [file 40942_2020_230_MOESM2_ESM.pdf]

Search Strategy	
**Method No.**	**Search terms**
PubMed	
1.	Tomography, Optical Coherence [Majr]
2.	Diabetic retinopathy [Majr]
3.	Retinal neovascularization [Majr]
4.	2 or 3
5.	Humans [MeSH Terms]
6.	English [lang]
7.	French [lang]
8.	Portuguese [lang]
9.	Spanish [lang]
10.	6 or 7 or 8 or 9
11.	1 and 4 and 5 and 10
PubMed	
1.	Optical coherence tomography [All Fields]
2.	OCT [All Fields]
3.	1 or 2
4.	Diabetic retinopathy [All Fields]
5.	DR [All Fields]
6.	Diabet* [All Fields]
7.	Retinopathy [All Fields]
8.	6 and 7
9.	4 or 5 or 8
10.	Neovascularization [All Fields]
11.	3 and 9 and 10
EMBASE	
1.	Optical coherence tomography /exp
2.	Retina neovascularization /exp
3.	Diabetic retinopathy /exp
4.	[humans] /lim
5.	[english] /lim
6.	[french] /lim
7.	[portuguese] /lim
8.	[spanish] /lim
9.	5 or 6 or 7 or 8
10.	1 and 2 and 3 and 4 and 9
EMBASE	
1.	Neovascularization :ab,ti
2.	Diabetic retinopathy :ab,ti
3.	Optical coherence tomography :ab,ti
4.	[humans] /lim
5.	[english] /lim
6.	[french] /lim
7.	[portuguese] /lim
8.	[spanish] /lim
9.	5 or 6 or 7 or 8
10.	1 and 2 and 3 and 4 and 9
