# Supplementary material for: Optical coherence tomography features of neovascularization in proliferative diabetic retinopathy: a systematic review
Source: Int J Retina Vitreous. 2020 Jun 29;6:26. doi: 10.1186/s40942-020-00230-3 (PMC7322867; doi:10.1186/s40942-020-00230-3)
Supplement: Supplementary file 3 — Additional file 3: Table S2. Summary of included studies. [file 40942_2020_230_MOESM3_ESM.docx]

| Study ID^[ref]^ | Study Center | Study Design | Included sample | NVD | NVE | IRMAs | Imaging technique | Outcomes |
| --- | --- | --- | --- | --- | --- | --- | --- | --- |
| Adhi 2016^[73]^ | USA | Prospective  cross-sectional | 88 diabetic eyes  (25 with PDR)  22 healthy control eyes | - | - | - | SS-OCT  SD-OCT  CFP  FA | Vitreoretinal interface in DR (bursa premacularis, area of Martegiani, PVD, vitreoschisis, vitreomacular adhesions, vitreous clumping)  PDR eyes had a thicker posterior hyaloid compared with normal and NPDR eyes, vitreoschisis 65% PDR eyes, pegs 55% PDR eyes |
| Akil 2019^[21]^ | Canada | Review | DR | - | - | - | OCTA | Characteristics of MA, NPA, FAZ, DME, IRMA and NVC (NVD and NVE), choroid |
| Akiyama 2018^[47]^ | Japan | Retrospective case-series | 17 eyes PDR | 17 eyes | - | - | SS-OCT  OCTA | Relationship between NVD and the PVD status  NVD arose from outside the cup and grew along the posterior hyaloid. Absence of PDV determinant to the growth of NVD |
| Arevalo 2013^[74]^ | USA  Saudi Arabia  Venezuela | Review | Retinal pathologies affecting the posterior pole  (including DR) | - | - | - | OCT | DME, PDR, features of subhyaloid hemorrhage, preretinal membranes and posterior hyaloid, TRD |
| Arya 2019^[58]^ | USA | Retrospective case-series | 14 eyes with severe NPDR and PDR | 26 lesions | | 70 lesions | WF SS-OCTA  FA | Distinguishing features of IRMA and NV using OCTA  OCTA demonstrated specificity of 99% and sensitivity of 92% in identifying IRMA and NV |
| Chatziralli 2016^[55]^ | UK | Retrospective case-series | 47 eyes with PDR | - | 47 eyes | - | SD-OCT  FA | Time to regression of NVE based on relative location to ILM following PRP |
| Cho 2013^[14]^ | USA | Retrospective cross-sectional | 16 eyes with PDR | 9 eyes | 6 eyes | 4 eyes | SD-OCT | Characterization of NVC, IRMA, and NVC-related traction with and without RD  Distinguishing features of IRMA and NV  Vitreoretinal changes associated with NVCs |
| Choi 2017^[59]^ | USA | Prospective  cross-sectional | 89 eyes from diabetic patients  (9 with PDR)  63 eyes from healthy controls | - | - | - | CFP  SS-OCTA  FA | Vascular abnormalities (clustered, dilated or tortuous capillaries and capillary loops)  Retinal capillary density, NPAs, FAZ, NV  PDR eyes had lower retinal capillary density compared with normal eyes |
| Cole 2016^[9]^ | USA  Brazil | Review | DR | - | - | - | CFP  Fundus autofluorescence  NIR image  FA  OCT  OCTA | MA, NVC, IRMA  Retinal traction  NPAs |
| Coscas 2018^[37]^ | France | Review | Healthy subjects  Diabetic patients | - | - | - | OCTA  FA | Characterization of MA, IRMA, venous beading, NVC, FAZ area and macular vascular density |
| De Barros Garcia 2017^[52]^ | Brazil | Review | DR | - | - | - | OCTA | MA, NVCs, IRMA  NPAs |
| De Carlo 2016^[24]^ | USA | Retrospective observational | 52 eyes with PDR | - | 13 eyes | 6 eyes | OCTA | Characterization of NVE and distinguishing features from IRMA, relation of NVE to retinal NPA and IRMA  92% eyes had NVE adjacent to retinal NPA and 50% were adjacent to IRMAs. |
| Elbendary 2018^[38]^ | Egypt | Cross-sectional | 32 eyes with PDR | 24 eyes | 8 eyes | 3 eyes | SS-OCTA  FA | Characteristics of NVC and IRMA  FAZ, NPA |
| Falavarjani 2019^[42]^ | Iran | Prospective interventional case series | 11 eyes with PDR | 11 eyes | - | - | OCTA | Time to regression of NVD following intravitreal bevacizumab; regression was observed as early as 24h |
| Gabr 2018^[35]^ | USA | Prospective interventional | 20 eyes with PDR | - | - | - | Intraoperative SS-OCT | Intraoperative detection of complications of PDR |
| Gajree 2017^[11]^ | UK | Review | DR | - | - | - | CFP  FA  OCT  OCTA | MA, NVCs  NPAs |
| Gildea 2019^[48]^ | UK | Review | DR | - | - | - | OCTA | Characteristics of MA, NVC, NPA, FAZ,  Vessel density and tortuosity  DME and macular ischemia |
| He 2019^[62]^ | China | Prospective pilot study | 44 eyes with PDR | - | 44 eyes | - | OCTA  FA | Changes in NVE size following PRP alone or with anti-VEGF  Combined treatment more effective in NVE regression |
| Hirano 2018^[57]^ | Japan | Prospective case-series | 37 eyes with DR  (13 with PDR) | - | - | - | WF SS-OCTA with EFI  CFP  FA | Comparison of SS-OCT with EFI to FA -NVC, NPA  SS-OCTA – sensitivity of 79% and specificity of 96% for NVC and of 96% and 100% for NPA |
| Hu 2019^[50]^ | China | Prospective observational trial | 24 eyes with PDR | - | - | - | OCTA | Quantification of change in neovascular pattern on fibrovascular membranes following intravitreal conbercept injection, vessel skeleton density and vessel density  OCT has potential to evaluate antiangiogenic effect in PDR |
| Hwang 2015^[22]^ | USA | Prospective case-series | 4 eyes with PDR | 3 eyes | 4 eyes | 1 eye | OCTA  FA | OCTA features of DR: FAZ size and shape, capillary dropout and arteriolar characteristics, MA and NVC |
| Hwang 2016^[72]^ | USA | Case-control | 47 eyes with DR (23 with PDR)  29 healthy eyes | - | - | - | OCTA | NVC, NPAs, capillary abnormalities |
| Ishibazawa 2015^[23]^ | Japan | Prospective case-series | 47 eyes with DR  (11 with PDR) | 5 eyes | - | - | CFP  FA  SD-OCT  OCTA | OCT in PDR: characterization of MA, IRMA, NVD, NVD flow area, NPA  Changes in NVC after anti-VEGF treatment |
| Ishibazawa 2016^[28]^ | Japan | Cross-sectional | 40 eyes with PDR | 25 eyes | 15 eyes | - | CFP  FA  SD-OCT  OCTA | Characterization of NVC in treatment-naïve or previously treated PDR using OCTA  Changes in NVC following PRP, NVC flow area  Description of EVP |
| Jia 2015^[41]^ | USA  Germany | Review  case-series | Healthy subjects  DR patients  AMD patients | - | - | - | FA  OCTA | Characterization of NVC and capillary dropout in DR |
| Kashani 2017^[53]^ | USA | Review | DR  Other pathologies | - | - | - | FA  ICG  SD-OCT  OCTA | Detection of NVC and IRMA using OCTA in DR  Staging of DR by OCTA  Study of capillary density in DR |
| Karst 2018^[71]^ | USA | Cross-sectional | 35 eyes with DR  (16 with PDR) | 7 lesions | | 20 lesions | Adaptive optics SLO  CFP  NIR image  SD-OCT | Characterization of MA, IRMA and NVC using AOSLO and comparison of findings with standard imaging techniques  Characterization of non-vascular lesions |
| Kim 2016^[75]^ | Korea | Retrospective case-series | 26 eyes with PDR | - | - | - | SD-OCT  Ultrasonic biometry | Morphology of tractional retinal elevation (TRE) in PDR  TRE may progress to TRD or tractional retinoschisis |
| Lee 2015^[16]^ | UK | Retrospective case-series | 12 eyes with DR  (9 eyes with PDR) | 2 eyes | 9 eyes | 4 eyes | FA  SD-OCT | Distinguishing features of IRMA and NVC using SD-OCT  IRMAs had hyperreflective dots in inner retina and ILM outpouching while NVE had ILM and/or posterior hyaloid breach and vitreous hyperreflective dots |
| Lee 2016^[44]^ | USA | Review | DR | - | - | - | CFP  FA  OCT  OCTA | MA, vascular abnormalities, NPA, FAZ, NV  Capillary perfusion density, vessel area, flow index,  PDR patients had significant lower vessel density values compared to NPDR patients |
| Liu 2018^[26]^ | China | Review | DR | - | - | - | OCTA | Characteristics of MA, DME, IRMA and NVC  Changes following PRP and anti-VEGF |
| Matsunaga 2015^[39]^ | USA | Cross-sectional | 47 eyes with DR  (13 with PDR) | 1 eye | - | - | FA  WF FA  SS-OCTA | OCTA of DR  Characteristics of cotton-wool spots, NPA, IRMA, intraretinal fluid, MA, NVD |
| Miura 2015^[19]^ | Japan | Prospective nonrandomized clinical trial | 17 eyes with PDR  8 eyes from healthy subjects | 12 eyes | 11 eyes | 1 eye | Prototype Doppler OCT | Characterization of NVC and IRMA using Doppler-OCT |
| Mizukami 2017^[78]^ | Japan | Retrospective case-series | 157 eyes with DR  11 eyes with PDR | - | - | - | SD-OCT | Number of hyperreflective foci in vitreous on SD-OCT increasing with severity of DR |
| Motulsky 2019^[43]^ | USA | Prospective case-series | 24 eyes with severe NPDR and PDR | 12 eyes | 22 eyes | - | CFP  SD-OCT  WF SS-OCTA | WF SS-OCT to detect and monitor NVC in PDR  Changes in NVC following anti-VEGF and PRP  Retinal perfusion |
| Muqit 2014^[15]^ | UK | Secondary analysis of two prospective clinical trials | 50 eyes with PDR | 33 eyes | 15 eyes | - | CFP  FD-OCT  WF-FA | FD-OCT characterization of NVD and NVE  Retinoschisis, vitreoretinal changes, traction retinal detachment |
| Muqit 2014^[36]^ | UK | Retrospective  case-series | 4 eyes with PDR | - | - | - | SS-OCT | SS-OCT characterization of NVCs, cortical vitreous and vitreoretinal interface  Tractional NVCs, distinguishing features of vitreoschisis and RD |
| Nesper 2017^[65]^ | USA | Review | DR | - | - | - | OCTA  Visible-Light OCT | Macular vessel density reduced in PDR patients  FAZ, non-perfusion areas, vascular flow, NV, DME, MA |
| Pan 2018^[25]^ | China | Cross-sectional | 35 eyes with PDR | 35 lesions | 75 lesions | 12 lesions | CFP  FA  OCTA | Evaluation of origin and morphology of NVC and IRMA  Quantification of NPAs  Classification proposal of NVE based on OCTA |
| Pierro 2017^[49]^ | Italy | Review | DR | - | - | - | CFP and infrared fundus photography  FA  OCT | Characteristics of DME, macular ischemia, MA and NVC  Preretinal and intravitreal hemorrhages  PVD, vitreoschisis, TRD  Choroidal thickness |
| Russell 2019^[31]^ | USA | Prospective case-series | 20 eyes with PDR | - | - | - | UWF FA  UWF fundus photography  SD-OCT  WF SS-OCTA | Characteristics of IRMA and NVC  Distinguishing features of MA, IRMA and NVC  Changes following PRP and anti-VEGF |
| Russell 2019^[32]^ | USA | Retrospective case-series | 651 eyes with PDR | 336  lesions | 587  lesions | - | UWF FA  WF SS-OCTA | Distribution of diabetic NVC |
| Sambhav 2017^[63]^ | USA | Review | DR  Other pathologies | - | - | - | OCTA  SS-OCTA | NV, FAZ area and DR severity  Capillary perfusion density and DR severity  Comparison of OCTA with FA |
| Savastano 2018^[20]^ | Italy | Case-series | 10 eyes with PDR | 10 eyes | - | - | FA  OCTA | Characterization of NVD (number, area, greatest linear dimension, morphology) and comparison with FA |
| Sawada 2018^[60]^ | Japan | Cross-sectional | 58 eyes with DR  (27 with PDR) | - | - | - | Wide-angle OCTA  UWF FA | Detection rates of NPA and NVC  Location of NVC |
| Schaal 2019^[30]^ | Switzerland | Cross-sectional | 120 eyes from diabetic patients  (19 with PDR) | - | - | 72 eyes | CFP  WF SS-OCTA | Good interrater agreement of retinal NPA, IRMAs and NVC. SS-OCT showed a higher detection of IRMAs |
| Schwartz 2020^[27]^ | UK | Retrospective case-series | 47 eyes with PDR | 41 eyes | 11 eyes | - | CFP  OCT  OCTA  UWF FA | Detection rates of new-onset, regression and reactivation of NVD, NVE and detection of progression to TRD  NVC regression after treatment |
| Stanga 2016^[46]^ | UK | Retrospective case-series | 86 eyes from diabetic patients | 28/28 eyes | 16/28 eyes | - | FA  SS-OCTA | FAZ, DME, hard exudates, microvascular abnormalities  NV, NPAs  OCTA non-inferior to FA for the study of the posterior pole |
| Tan 2016^[16]^ | Singapore | Review | DR | - | - | - | UWF CFP  FA  FAF  OCT  OCTA  SS-OCT | Assessment of DR severity  NVD, MA, NPAs, DME  Rates of vitreoschisis  FAZ enlargement  Intraoperative use of OCT |
| Tran 2018^[61]^ | Canada | Review | DR | - | - | - | CFP  FA  OCT  OCTA  Adaptive optics | Clinical applications of various imaging modalities in DR  FAZ, NPA, MA, edema, NVC |
| Tran 2019^[64]^ | USA | Review | DR | - | - | - | OCTA | Applications of OCTA in managing DR  Vascular density and DR severity  MA, FAZ, IRMAs, NVCs, NPAs, DME |
| Vaz-Pereira 2016^[17]^ | UK  Portugal | Cross-sectional | 43 eyes with PDR | 23 lesions | 38 lesions | - | CFP  FA  SD-OCT | Morphologic evaluation of NVCs and vitreoretinal and retinal parameters to find distinguishing features of active and quiescent NVC |
| Vaz-Pereira 2017^[18]^ | Portugal  USA | Cross-sectional | 51 eyes with PDR | - | - | - | CFP or red-free fundus photography  NIR images  SD-OCT  FA | Relationship between NVCs and the posterior vitreous cortex and spaces (premacular bursa, prevascular fissures and perimacular cisterns)  NVC morphology  Vitreoschisis, PVD, vitreous and subhyaloid hemorrhage |
| Wylegala 2016^[54]^ | Poland | Review | DR  Other pathologies | - | - | - | FA  OCTA  SD-OCT  SS-OCT | Comparison of FA to OCTA, regarding their sensitivity to detect and/or characterize vascular abnormalities, NPAs,  FAZ area, CNV, and response to anti-VEGF therapy |
| You 2020^[70]^ | USA | Prospective case-series | 27 patients with clinical NPDR | 4 eyes | | - | CFP  WF SS-OCTA | Detection of clinically unsuspected NVC using WF SS-OCT NVC area |
| Yu 2016^[56]^ | China | Prospective case-series | 50 patients with retinal disease  (10 with PDR) | - | - | - | FA  ICG  OCTA | MA, FAZ, NPA, NVC |
| Zhang 2015^[40]^ | USA | Case-control | 20 patients with DR (10 with PDR)  8 patients with AMD  9 healthy controls | - | - | - | OCTA | Imaging processing of 3D-OCT  NVC, retinal ischemia, macular edema |
| Zhang 2016^[51]^ | USA | Prospective case-series | 6 cases  of retinal disease including DR (1 eye PDR) | 1 eye | 1 eye | - | CFP  FA  SD-OCT  WF OCTA | Imaging capabilities in evaluating the vitreous cavity, retinal layers, RPE, and choriocapillaris  NVD, NVC flow, macular capillary perfusion  WF OCTA compared to FA |
| Zhang 2018^[29]^ | USA | Case-series | 3 patients with PDR | - | - | - | SS-OCTA  UWF SS-OCTA | UWF-OCTA compared to 50º FA and conventional OCTA  NV, vessel density, NPA maps |
| Zhang 2018^[40]^ | China | Prospective  case-series | 15 eyes with PDR | 15 eyes | - | - | CFP  SD-OCT  OCTA | NVD changes following intravitreal conbercept injection and PRP |

*CFP* color fundus photography, *DME* diabetic macular edema, *DR* diabetic retinopathy, *EFI* extended field imaging, *EVP* exuberant vascular proliferation, *FA* fluorescein angiography, *FAZ* foveal avascular zone, *FD-OCT* Fourier-domain optical coherence tomography, *ILM* inner limiting membrane, *IRMA* intraretinal microvascular abnormalities, *MA* microaneurysm, *NIR* image near-infrared reflectance image, *NPA* retinal nonperfusion areas, *NVC* neovascular complex, *NVD* neovascularization of the disc, *NVE* neovascularization elsewhere, *OCT* optical coherence tomography, *OCTA* optical coherence tomography angiography, *PDR* proliferative diabetic retinopathy, *PRP* panretinal photocoagulation, *PVD* posterior vitreous detachment, *SD-OCT* spectral-domain optical coherence tomography, *SS-OCT* swept-source optical coherence tomography, *SS-OCTA* swept source optical coherence tomography angiography, *TRD* tractional retinal detachment, *VEGF* vascular endothelial growth factor, *VH* vitreous hemorrhage, *UWF* ultra-widefield, *WF* widefield
